# Assessment of Brix values for estimating nitrogen and gross energy concentrations of pig urine: a technical note

**DOI:** 10.5713/ab.24.0684

**Published:** 2025-02-27

**Authors:** Jeonghyeon Son, Hyunseok Do, Beob Gyun Kim

**Affiliations:** 1 Department of Animal Science, Konkuk University, Seoul, Korea; 2 Current address: Department of Animal Science, North Carolina State University, Raleigh, NC, USA

**Keywords:** Brix, Gross Energy, Models, Nitrogen, Swine, Urine

## Abstract

The present study aimed to develop equations for estimating the dry matter (DM), nitrogen (N), and gross energy (GE) concentrations in pig urine using Brix values. A total of 96 urine samples were obtained from pigs of 6.0 to 62.2 kg initial body weight fed 12 diets in three experiments. The animals were fed a corn-soybean meal-based diet or a corn diet. The N concentrations in the urine samples ranged from 0.12% to 2.06% and the GE concentrations ranged from 2 to 187 kcal/kg. The urine Brix values were positively correlated with urinary DM, N, and GE concentrations (r = 0.94, 0.87, and 0.93, respectively; p<0.001). The models for estimating urinary DM, N, and GE concentrations were: urinary DM (%) = 0.590×Brix+0.315 (r^2^ = 0.91; p<0.001); urinary N (%) = 0.0798×Brix+ 0.148 (r^2^ = 0.84; p<0.001); urinary GE (kcal/kg) = 11.96×Brix+2.33 (r^2^ = 0.87; p<0.001), where the Brix value of urine is expressed as %. Overall, the Brix values correlated positively with the DM, N, and GE concentrations in pig urine and can thus be used for estimating the concentrations of these urinary components.

## INTRODUCTION

Brix is a unit that represents the quantity of dissolved solids (g) in 100 grams of a solution. The Brix refractometer enables convenient and instant analyses of solid concentrations dissolved in a solution. Brix has been widely used to estimate the sugar content in fruits [1]. In addition, Brix has also been employed as an indicator of non-sugar soluble solid concentrations in liquids [1]. In the field of animal science, researchers have utilized Brix for estimating the concentration of immunoglobulins in sow and cattle colostrum [2,3].

Recently, the application of the Brix measurement has been extended to the estimation of chemical components in animal-derived liquid samples, such as saliva [4]. In this experiment, Kessler et al [4] reported positive correlations between dry matter (DM) concentrations and Brix values of bovine urine. As the primary solid components of urine are nitrogen (N)-containing compounds, such as urea and creatine [5,6], N and gross energy (GE) concentrations in urine could also be estimated based on Brix values. However, models for estimating urinary N and GE concentrations in pigs using urine Brix values have not yet been developed. Therefore, the objective of this study was to develop equations for estimating the urinary DM, N, and GE concentrations in pigs using Brix values.

## MATERIALS AND METHODS

The protocols for animal experiments to obtain urine samples were approved by the Institutional Animal Care and Use Committee of Konkuk University (Seoul, Korea; KU22046 and KU23055).

### Animals, diets, and experimental design

A total of 96 urine samples were obtained from pigs fed 12 diets in 3 digestibility experiments [7,8]. All experiments were conducted using crossbred barrows (Landrace×Yorkshire) under the same environmental conditions. At the beginning of the animal experiments, the average initial body weight (BW) of the pigs in Exp. 1, 2, and 3 was 7.0, 29.9, and 50.2 kg, respectively (standard deviation = 0.8, 1.8, and 2.2). The animals were individually housed in metabolism crates equipped with a feeder, a fully slatted floor, and a urine tray, facilitating the total, but separate collection of urine and feces from each pig. In all experiments, the animals were fed corn-soybean meal-based diets or a corn diet.

### Feeding and sample collection

In Exp. 1, the daily feed allowance was calculated as 5.0% of initial BW and divided into three equal meals provided at 08:00, 12:30, and 17:00 h. In Exp. 2 and 3, the daily feed allowance was calculated as 4.5 and 4.0% of initial BW of pigs, respectively, and divided into two equal meals provided at 08:00 and 17:00 h. Each period of Exp. 1 consisted of a 4-day adaptation period and a 4-day collection period. Each period of Exp. 2 and 3 consisted of a 5-day adaptation period and a 4-day collection period. In Exp. 1, individual pig’s urine was collected in a bucket containing 6 *N* HCl for N preservation from day 5 at 14:00 h to day 9 at 14:00 h continuously. On day 9, urine samples were obtained for each pig. The amount of 6 *N* HCl was determined based on the initial BW of pigs in each period [9]. In Exp. 2 and 3, urine collection from individual pigs was initiated at 10:00 h on day 6 and terminated at 10:00 h on day 10. Urine was weighed at 10:00 h and 19:00 h and a 10% subsample was stored at −20°C immediately after collection due to the absence of acid addition. At the end of the collection period, the frozen urine samples were thawed and pooled to obtain urine samples for chemical analysis. The urine samples were filtered using a cotton sheet.

### Chemical analyses

The 0.2 ml of urine samples was analyzed for Brix percentage using a digital refractometer (SUGAR-1 PLUS; CAS Inc., Yangju, Korea). For the analysis of urinary DM and GE, 3 mL of thawed urine sample was soaked into a cotton ball and lyophilized [10]. After weighing the lyophilized cotton balls containing urine solids to determine the urinary DM, the lyophilized cotton balls with urine were analyzed for GE using a bomb calorimetry (Parr 1261 bomb calorimeter; Parr Instruments Co., Moline, IL, USA). The urinary N concentration was determined using a digestion apparatus and an automatic distillation unit (method 990.03; Büchi Labortechnik AG, Flawil, Switzerland) as described by AOAC [11]. The ammonia collected was titrated using the standard 0.1 *M* HCl. All chemical analyses were performed in duplicate. Two urine samples from Exp. 3 with negative GE values were excluded from the dataset.

### Statistical analyses

The correlation coefficients (r) among the Brix value and chemical components in the 94 urine samples were determined using the CORR procedure of SAS (SAS Institute Inc., Cary, NC, USA). The equations for estimating the urinary DM, GE, and N concentrations were developed using the REG procedure of SAS, with the urinary Brix value as the independent variable. Ten observations with Cook’s distance greater than Fox’s criterion (>0.043) were considered outliers and excluded, resulting in 84 observations in the final model. The root mean square error and determination coefficient (r^2^) were used as indicators of the accuracy of the developed models. The alpha level used for determining statistical significance was less than 0.05.

## RESULTS

The Brix values of the urine samples and urinary DM, N, and GE concentrations ranged from 0.55% to 14.20%, 0.42% to 8.75%, 0.12% to 2.06%, and 2 to 187 kcal/kg, respectively (Table 1). The Brix values in urine were positively correlated (p<0.001) with the urinary DM (r = 0.94), N (r = 0.87), and GE (r = 0.93) concentrations (Table 2).

**Figure 1:**
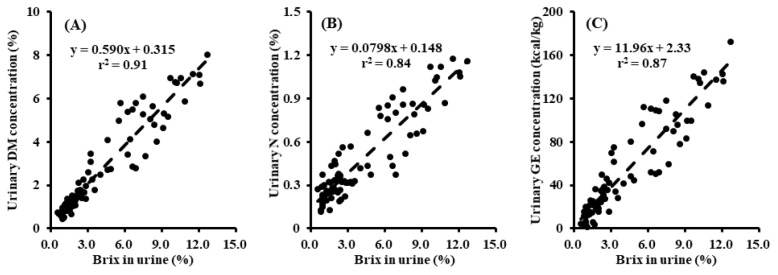
Equations for estimating urinary dry matter (DM), nitrogen (N), and gross energy (GE) concentrations on the basis of the urine Brix value (%) of pigs (n = 84). (A) Urinary DM (%) = 0.590×Brix+0.315, with root mean square error (RMSE) = 0.686, r^2^ = 0.91, and p<0.001. (B) Urinary N (%) = 0.0798×Brix+0.148, with RMSE = 0.121, r^2^ = 0.84, and p<0.001. (C) Urinary GE (kcal/kg) = 11.96×Brix+2.33, with RMSE = 15.76, r^2^ = 0.87, and p<0.001. The urine Brix value is expressed as a percentage (%).

The equations for estimating urinary DM, N, and GE concentrations in pigs were developed using urine Brix values (Figure 1): urinary DM (%) = 0.590×Brix+0.315 (r^2^ = 0.91; p<0.001); urinary N (%) = 0.0798×Brix+0.148 (r^2^ = 0.84; p<0.001); urinary GE (kcal/kg) = 11.96×Brix+2.33 (r^2^ = 0.87; p<0.001). All independent variables and the intercepts were significant (p<0.05) with the exception of the intercept of the equation for GE estimation. Urine Brix values are expressed as percentage.

## DISCUSSION

Pigs excrete absorbed but unutilized energy and nutrients through urine [9]. Urinary N excretion accounts for 20.4 to 49.4% of N intake of pigs [7,8,12]. Energy excretion through urine also accounts for a considerable portion of the GE intake in pigs [7,9]. Therefore, an accurate determination of urinary N and GE concentrations is critical in experiments concerning N and energy balance. However, the direct determination of urinary N concentrations is a time-consuming and laborious process, and that of GE concentrations is also labor-intensive because it involves the lyophilization of urine samples [10]. To reduce time for analyzing urinary GE, the models for estimating urinary GE have been developed using urinary N as an independent variable in previous studies [13,14]. The present study aimed to develop models for estimating urinary N and GE concentrations using an independent variable of urine Brix that can be measured instantly.

Although the variability of Brix values, nutrient concentrations, and GE of urine among 3 experiments is not the major interest of the present study, the large variability makes the equations developed appliable to a wide range of urine. The mean values of Brix, nutrients, and GE of urine from Exp. 1 were greater than those obtained from Exp. 2 and 3, which is likely due to the amino acid imbalance of the diets used for Exp. 1. Crystalline amino acids were used to meet the requirements for indispensable amino acids in Exp. 2 and 3, whereas the diets in Exp. 1 were formulated to determine the energy values of the ingredients with varying protein concentrations. Thus, the dietary protein concentrations in Exp. 1 varied with excessive or deficient amino acids.

The high correlation between the urine Brix values and urinary DM concentrations observed in the present study is in agreement with the results from a dairy cow study [4]. This high correlation is reasonable, as the Brix value is an index of refraction, which is largely affected by the quantity of solids dissolved in a solution. Similarly, urine Brix values were highly correlated with urinary N and GE concentrations as well likely because the urinary solids consist mainly of urea, creatine, and ammonium ions that contain N and energy [5,6]. Considering that urine Brix values are highly correlated with the urinary DM, N, and GE concentrations, the Brix value can be used as an independent variable for estimating the urinary chemical components.

To the best of our knowledge, this is the first study to report equations for estimating DM, N, and GE concentrations in pig urine using Brix values. Similarly, an model for estimating urinary DM concentrations of dairy cows using urine Brix values was developed using 40 urine samples [4]. Although the species are different, the r^2^ value of the present model for estimating the urinary DM concentration is comparable (0.91 vs. 0.88) to that of the of the model by Kessler et al [4]. Although an anecdotal observation, the present equation for estimating urinary GE using urine Brix values had less residual standard deviation (16 kcal/kg) compared with a previous equation for estimating urinary GE using urinary N concentrations with a residual standard deviation of 54 kcal/kg [15]. This indicates that urine Brix values compared with urinary N may be a better independent variable for estimating urinary GE. However, because the correlation between the urinary N and GE concentrations was also strong (r = 0.92) in the present study, further research on this relationship is warranted.

## CONCLUSION

The DM, N, and GE concentrations are correlated with Brix values in urine. The equations with Brix value as an independent variable can be used for estimating the urinary DM, N, and GE concentrations in pigs.

## Figures and Tables

**Table 1:** Range and variability of Brix values and chemical component concentrations in urine samples and body weight of pigs^1)^

Item	n	Mean	Minimum	Maximum	SD	CV
Experiment 1						
Brix value (%)	48	7.95	1.30	14.20	3.10	38.9
Dry matter (%)	48	5.08	1.36	8.75	1.98	39.0
Nitrogen (%)	48	0.84	0.22	2.06	0.38	45.6
Gross energy (kcal/kg)	48	98	15	187	45	45.5
Body weight (kg)	48	9.4	6.0	16.3	2.3	24.8
Experiment 2						
Brix value (%)	24	1.77	0.80	3.20	0.71	40.0
Dry matter (%)	24	1.38	0.55	3.08	0.61	44.4
Nitrogen (%)	24	0.25	0.12	0.38	0.07	30.0
Gross energy (kcal/kg)	24	29	5	75	17	59.7
Body weight (kg)	24	33.5	27.8	40.9	4.2	12.7
Experiment 3						
Brix value (%)	22	1.63	0.55	2.85	0.65	40.0
Dry matter (%)	22	1.14	0.42	2.24	0.48	42.0
Nitrogen (%)	22	0.33	0.15	0.56	0.11	33.7
Gross energy (kcal/kg)	22	18	2	46	13	75.4
Body weight (kg)	22	54.7	48.5	62.2	4.6	8.3

1) The body weight was obtained from previous experiments [7,8].

SD, standard deviation; CV, coefficient of variation.

**Table 2:** Correlation coefficients among the Brix value and chemical components in urine samples from pigs^1)^

Item	Dry matter	Nitrogen	Gross energy
Brix	0.94***	0.87***	0.93***
Dry matter	-	0.93***	0.99***
Nitrogen	-	-	0.92***

1) A pig was considered as an experimental unit (n = 94).

*** p<0.001.
